# Cation Diffusion Facilitators Transport Initiation and Regulation Is Mediated by Cation Induced Conformational Changes of the Cytoplasmic Domain

**DOI:** 10.1371/journal.pone.0092141

**Published:** 2014-03-21

**Authors:** Natalie Zeytuni, René Uebe, Michal Maes, Geula Davidov, Michal Baram, Oliver Raschdorf, Merav Nadav-Tsubery, Sofiya Kolusheva, Ronit Bitton, Gil Goobes, Assaf Friedler, Yifat Miller, Dirk Schüler, Raz Zarivach

**Affiliations:** 1 Department of Life Sciences, Ben Gurion University of the Negev, Beer-Sheva, Israel; 2 National Institute for Biotechnology in the Negev, Ben Gurion University of the Negev, Beer-Sheva, Israel; 3 Ludwig Maximillian University of Munich, Department of Biology I, Martinsried, Germany; 4 Institute of Chemistry, The Hebrew University of Jerusalem, Givat Ram, Jerusalem, Israel; 5 Department of Chemistry, Ben Gurion University of the Negev, Beer-Sheva, Israel; 6 Ilse Katz Institute for Nanoscale Science and Technology, Ben Gurion University of the Negev, Beer-Sheva, Israel; 7 Department of Chemistry, Bar-Ilan University, Ramat Gan, Israel; 8 Department of Chemical Engineering, Ben Gurion University of the Negev, Beer-Sheva, Israel; University of South Florida College of Medicine, United States of America

## Abstract

Cation diffusion facilitators (CDF) are part of a highly conserved protein family that maintains cellular divalent cation homeostasis in all domains of life. CDF's were shown to be involved in several human diseases, such as Type-II diabetes and neurodegenerative diseases. In this work, we employed a multi-disciplinary approach to study the activation mechanism of the CDF protein family. For this we used MamM, one of the main ion transporters of magnetosomes – bacterial organelles that enable magnetotactic bacteria to orientate along geomagnetic fields. Our results reveal that the cytosolic domain of MamM forms a stable dimer that undergoes distinct conformational changes upon divalent cation binding. MamM conformational change is associated with three metal binding sites that were identified and characterized. Altogether, our results provide a novel auto-regulation mode of action model in which the cytosolic domain's conformational changes upon ligand binding allows the priming of the CDF into its transport mode.

## Introduction

Divalent metal cations are essential elements for proper cellular development and function [1]. Within the cells, metal cation concentrations are tightly regulated as their excess accumulation can lead to cytotoxicity [2]. Therefore, diverse sensory and export systems have evolved to sustain cellular homeostasis. One group of metal ion efflux transporters that regulates metal ion homeostasis and can be found in all domains of life is the Cation Diffusion Facilitator (CDF) protein family [3]. Members of the CDF family usually transport cytoplasmic divalent metal cations, including Cd, Co, Fe, Mn, Ni and Zn, by exploiting the proton motive force [4]–[8]. CDFs are divided into three substrate-specific clades: Zn-CDF, Fe/Zn-CDF and Mn-CDF [9]. CDFs broad substrate spectrum explains their participation in diverse cellular processes taking place at different cellular compartments, such as the vacuolar membranes of plants and yeast, the Golgi apparatus of animal cells or the bacterial cell membrane [10]. The association of altered regulation or mutations within several human CDFs (ZnT or *SLC30A* 1–10) with severe human diseases demonstrates the central role of CDFs in cellular metal homeostasis [11]–[14]. For example, the increased risk of acquiring Type-II diabetes is associated with a single amino acid polymorphism in the human ZnT-8 [15].

All CDF transporters share a common two-domain fold that contains a transmembrane domain (TMD) and a cytosolic C-terminal domain (CTD) [3]. The structure of FieF (YiiP) from *Escherichia coli*, which belongs to the Fe/Zn-CDF clade, was determined in a zinc-bound active form and found to present a homo-dimeric fold [16]. Other structural studies of CDFs have mainly focused on the soluble cytosolic domain. These determined CTD structures include the Zn-bound and apo-forms of CzrB protein from *Thermus thermophilus*, as well as the apo-form of TM0876 protein from *Thermotoga maritime* [17], [18]. Although the CTDs show a high degree of sequence variability between different species, all available CTD structures share a similar metallochaperone-like fold. This fold is a common structural module that is often involved in cytoplasmic metal trafficking and transport by forming a metal donor-acceptor interface with the relevant transporting machinery [19]. A significant flexible movement within the metallochaperone-like CzrB dimer was induced upon zinc binding and is considered to be associated with transport regulation [18]. However, based on fluorescence resonance energy transfer experiments, recent work done on FieF suggested a CDF-activating mechanism [16] which is inconsistent with the CzrB results. Although the CzrB and FieF CTDs share a common fold, the stable dimerization interface in CzrB was suggested to be dissociated in the FieF activation model upon zinc release. These discrepancies hinder our understanding of the CDF-activation mechanism and thus can hamper our understanding of CDF-related human diseases [20].

Magnetotactic bacteria (MTB) are a unique group of prokaryotes that utilize CDF proteins for the biomineralization of magnetic iron mineral nanoparticles. This bacterial biomineralization occurs within intracellular membrane-enclosed compartments called magnetosomes that serve as a geomagnetic navigation sensor [21], [22]. The genetically tractable alphaproteobacterium *Magnetospirillum gryphiswaldense* MSR-1 and its close relatives synthesize magnetic iron oxide – magnetite (Fe_3_O_4_) – nanocrystals that are aligned as intracellular chains. Magnetite biomineralization is controlled by a large set of proteins that are mostly associated with the magnetosome membrane (MM) [23], [24]. One of the most highly conserved and abundant integral MM proteins is the CDF transporter MamM. MamM was recently proposed to function as a magnetosome-directed iron transporter required for iron biomineralization since its deletion abolished magnetite biomineralization and mutation of residues within the conserved TMD metal-binding site resulted in alterations of magnetite crystal size, morphology and even mineral phase [25]. Furthermore, these *in vivo* studies demonstrated the ability of MamM to form dimers, as well as to stabilize MamB another CDF transporter of the MM [25].

To elucidate the discrepancies in the CDF activation models we initiated an in-depth protein structure-function analysis. In this article, we provide a new perspective for the CDF iron transporter MamM and propose a new auto-regulation mode of action model for the CTD of CDFs.

## Results

### MamM-CTD forms ‘V-shape’ stable dimers

**Figure 1:**
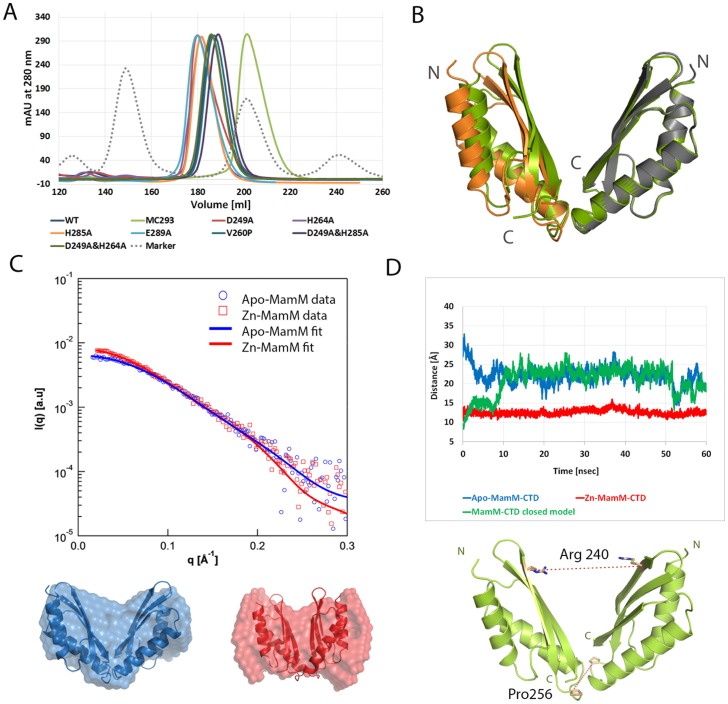
Apo-MamM-CTD forms stable dimers in solution. (A) Size-exclusion chromatography (Superdex200) of apo-MamM-CTD and different mutants elute in a volume appropriate for dimers. Calculated molecular weight form calibration curve and predicted molecular weight are ∼21 kDa/23 kDa for MamM-CTD 215–318 and ∼15 kDa/18 kDa for MamM-CTD 215–293 dimers. Molecular marker of Conalbumin (75 kDa), Ovalbumnin (43 kDa), Ribonuclease A (13.7 kDa) and Aprotinin (6.5 kDa) in dashed grey line. (B) Overall apo-MamM-CTD dimeric structures adopt the typical CDF-CTD fold presenting a flexible movement at the dimers' N-terminal region that faces the membrane: C222_1_ crystal form which contains a single monomer in the asymmetric unit (orange), the symmetry related monomer comprising the dimer (gray) and the F23 crystal form which contains a dimer in the asymmetric unit (green). (C) Small-angle X-ray scattering of MamM-CTD in solution with (red) and without (blue) added zinc. *Top* - The scattering data are shown as individual data points and the data fit as solid lines. *Bottom* - Calculated protein envelope from the scattering curves in surface representation. Superposition of these protein envelopes with determined apo-structure (blue) and the zinc bound predicted model (red) in cartoon representation. (D) Molecular dynamic simulations of MamM-CTD suggesting that MamM dimers are stable and that the N-terminal region of the dimer undergoes conformational changes upon zinc binding. The distance between the pair of Arg240-Cα from each monomer comprising the dimer was monitored through 60 nsec simulation. Blue – Apo-MamM-CTD displaying flexible fluctuations. Red – MamM-CTD predicted closed model with bound zinc at the central putative binding site displaying smaller and stable Arg240-Cα distance. Green – MamM-CTD predicted closed model without zinc adopts the apo-MamM-CTD flexible fluctuations.

To characterize the dimeric structure of MamM-CTD (residues 215-318) several independent experimental methodologies were employed. These included size exclusion chromatography, X-ray crystallography, Small Angle X-ray Scattering (SAXS) and Molecular Dynamics (MD) simulations. According to size-exclusion chromatography, MamM-CTD forms a stable dimer in solution (Fig. 1A), similar to other cytosolic domains of CDFs. The purified apo-MamM-CTD was crystallized and its structure was determined in two crystal forms (Table S1 and Fig. A in file S1). Each MamM monomer (residues 215–293) adopts the metallochaperone-like typical fold of CDF CTDs (Fig. 1B) whilst the C-terminal tail (residues 294–318) is disordered and could not be traced in the electron density map. To validate that the disordered C-terminal tail have no effect on the domain fold, we determined the structures of a C-terminal truncation MamM-CTD mutant (residues 215–293) that adopted a similar CTD fold (Table S1 and Fig. A in file S1). The dimeric MamM-CTD structures present a typical V-shaped assembly with some extent of flexibility at the top of the dimer (Fig. 1B) and with a stable dimerization interface located at the bottom of the dimer. SAXS measurements of MamM also confirmed that a V-shaped envelope was the best representation of the protein structure in solution, providing an additional support to the formation of stable dimers by the MamM-CTD (Fig. 1C). To further explore the extent of the observed flexibility at the top of the V-shaped dimer we performed MD simulations. We monitored the dimer behavior during a 60 nsec simulation with the MamM-CTD determined structure serving as an input. Analysis of these simulations included distance measurements between the Cα atoms of two pairs of identical residues from each monomer and Cα atoms of four residues to monitor the dihedral angle. The first pair was Arg240-Arg240 located at the top of the V-shaped dimer, while the second pair was Pro256-Pro256 located at the dimer interface at the bottom of the V-shape (Fig. 1D). The MD simulations analysis suggested that the bottom of the V-shaped structure is highly stable and rigid, whilst the angle between the monomers tends to change and permits the observed flexibility at the top of the V-shaped dimer (13-37 Å, Fig. 1D and Fig. B in file S1). Therefore, from the MD simulations one can propose that the dimer samples multiple conformations while searching for proper metal ions. Moreover, surface charge distribution of the MamM-CTD determined structure demonstrates that the two "arms" of the V-shaped dimer are negatively charged. The significant motions observed in MD simulations may be due to major charge repulsion between the two “arms” (Fig. C in file S1). Overall, the different structural states in the crystal are in line with MamM-CTD's flexibility that adopts different conformers. One can propose that the relatively low barriers between the different conformers allow more sampling of the conformers via domain motions around the hinge at the bottom of the V-shaped structure. The extent of this domain movement can substantially be reduced upon binding of divalent cations, as described in the following section.

### The MamM-CTD divalent cation binding induces conformational changes

Performing isothermal titration calorimetry (ITC) experiments with low protein concentrations allowed us to obtain the thermodynamic parameters of the MamM-CTD divalent cation binding. These experiments included the titration of divalent cations into a solution of the MamM-CTD at pH = 8.0 or pH = 5.7 to vary the charge state of imidazole rings in histidine residues. As iron is unstable at this pH range due to the formation of insoluble iron hydroxides, we mostly used stable zinc ions for the described *in vitro* experiments. Accordingly, MamM-CTD dimers bind four zinc ions with an affinity of 16±4 μM (Table 1; Fig. D in file S1) in alkaline buffer, as described for the FieF protein [26]. Altering the pH level to 5.7 abolished the binding and changed the reaction from exothermic to endothermic (Fig. D in file S1). The observed pH dependence suggests the participation of histidine residues in metal cation binding.

**Table 1:** Thermodynamics of zinc binding to MamM-CTD dimer and mutants as measured by Isothermal Titration Calorimetry.

Protein	N*	K_d_	ΔH	ΔS	ΔG
		(μM)	(kcal/mol)	(cal/mol/deg)	(kcal/mol)
MamM-CTD	4.1±0.2	16±4	−1.8±0.1	16	−4.77
MamM-CTD D249A	5.3±0.2	20±3	−1.68±0.07	15.4	−4.95
MamM-CTD H264A	4.8±0.2	20±3	−1.68±0.08	15.8	−4.71
MamM-CTD H285A	5.17±0.09	41±4	−1.42±0.03	15.3	−4.56
MamM-CTD E289A	2.0±0.2	21±6	−3.9±0.5	8.3	−2.48
MamM-CTD V260P	1.6±0.2	42±16	−3.4±0.5	7.68	−2.29
MamM-CTD D249A-H264A	-	-	-	-	-
MamM-CTD D249A-H285A	1.8±0.2	12±5	−1.3±0.2	18.1	−5.40
MamM 215-293	3.55±0.04	10±1	−2.78±0.04	13.6	−4.06

Data was fitted to the single-site binding isotherm using ORIGIN 7.0 software.

**Figure 2:**
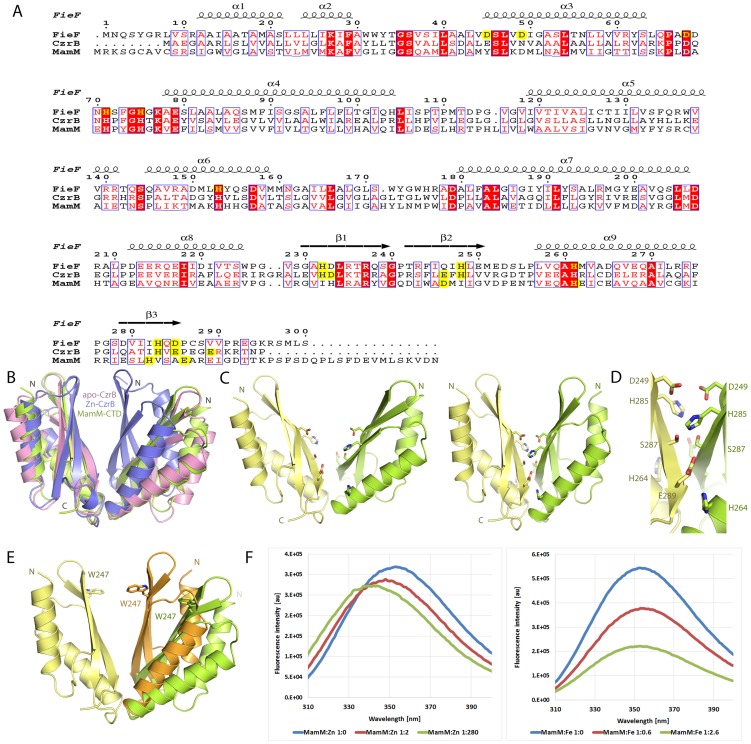
Divalent cation binding induce conformational change. (A) Multiple sequence alignment of MamM, FieF and CzrB proteins. Secondary structure is presented according to FieF structure. Residues that participate in zinc binding are highlighted in yellow. (B) MamM-CTD (green) displays open dimer conformation similar to apo-CzrB (pink) (Cα–RMSD = 1.19) and not to the closed Zn-CzrB (blue) protein conformation. (C) MamM-CTD dimer conformations in cartoon representation; residues predicted to participate in divalent cation binding are displayed in sticks representation. *Left*- Apo-MamM-CTD dimer conformation as determined by X-ray crystallography. *Right* – Zn/Fe bound MamM-CTD closed dimer conformation generated by overlaps of MamM-CTD monomers with determined structures of Zn-bound CDFs. (D) Divalent cation putative binding sites as displayed in the closed state MamM-CTD model. The central putative binding site involves Asp249 and His285 from each monomer. His264 and Glu289 from different monomers give rise to two peripheral and symmetrical putative binding sites. (E) MamM-CTD divalent cation binding-induced conformational changes alter the location of a single Trp residue between the apo (green) and cation-bound (orange) states. (F) Alterations in the natural florescence of Trp induced by divalent cation binding. *Left*- fluorescence peak wavelength shift upon zinc binding. *Right* – fluorescence peak quenching upon iron binding.

Overlaying the CTD structures of MamM and CzrB revealed that the apo form of MamM-CTD is similar to the apo-form of CzrB [18] (Fig. 2A). To determine whether the MamM-CTD can undergo similar conformational changes to the CzrB-CTD upon divalent cation binding, we tried to crystallize MamM-CTD in the presence of zinc, iron, cadmium, manganese and other metals. Despite numerous attempts and employing various crystallization techniques, cation-bound MamM-CTD crystals were not produced; instead, severe precipitation was encountered at the high protein concentration which precluded single crystal diffraction data.

Alternatively, we used a computational approach to obtain a structural model of the divalent cation-bound state of MamM (i.e. the closed state) by overlapping MamM-CTD monomers with the zinc bound state of CzrB (Fig. 2B). Examining this closed state model, three putative binding sites were revealed, in which Asp, Glu and His residues from the two monomers are brought into close proximity and may bind divalent cations. These putative binding sites are located at a unique location at the center of the V-shaped dimer as well as in a symmetrical manner on the periphery of the dimer (Fig. 2C–D). The central binding site is presumed to be the main site as it displays tight packing and contains two symmetry-related His285 and two Asp249 residues. The other two symmetrical peripheral binding sites contain His264 and Glu289 residues but no designated binding cavity was found. MD simulations were performed in order to examine the stability of the closed state model. Based on the ITC results (Table 1), four zinc ions were docked to the closed-state model as initial structures for the simulations: two at the putative central binding site and one in each peripheral site. Following the previously described residue distances and dihedral angles we found that the zinc-bound closed state model is highly stable throughout the 60 nsec simulation and maintains a 13 Å distance between the Cα of Arg240-Arg240 located at the top of the V-shaped dimer (Fig. 1D and Fig. B in file S1). When the closed-state model was used without the docked zinc ions, the dimer regained its flexible “arm” motions after 9 nsec (Fig. 1D and B in file S1). Thus, the binding of a divalent cation at the central putative binding site stabilizes MamM-CTD structure and supports our crystallography's closed-state model.

Experimental evidence for the MamM-CTD conformational changes were obtained from SAXS, Trp fluorescence and solid-state Nuclear Magnetic Resonance (ssNMR). Comparison between the SAXS-calculated protein envelopes of the non-bound and zinc-bound states of MamM-CTD suggested that the apo-protein undergoes conformational changes upon zinc binding, as the determined apo-structure no longer fits appropriately to the calculated envelope (Fig. 1C). Additional support was obtained using natural tryptophan fluorescence measurements, utilizing the single Trp residue in each MamM-CTD monomer. Trp247 is located at the top of the V-shaped dimer facing the central putative binding site (Fig. 2E). According to the MD simulations, the distance between the Cα of Trp247-Trp247 in the apo state ranges between 14–32 Å and 10–13 Å in the zinc-bound state (Fig. B in file S1). Introduction of increasing concentrations of zinc ions caused a fluorescence peak shift associated with increased hydrophobicity (Fig. 2F). Moreover, the natural Trp fluorescence can be quenched and result in a lower signal intensity by the binding of iron in close proximity to the Trp's indole ring. When increasing concentrations of iron ions were introduced to the MamM-CTD solution, a decrease in Trp fluorescence intensity was observed (Fig. 2F). This decrease suggests that the residues taking part in binding iron ions are in close proximity to Trp247 residue, meaning that they reside in the putative central binding site.

**Figure 3:**
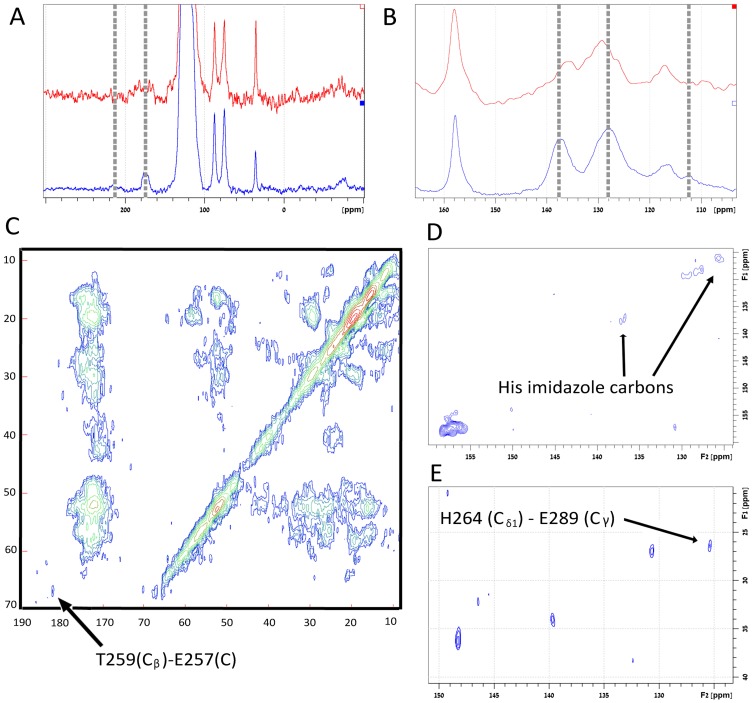
(A) ^15^N NMR spectra of [U-^13^C,^15^N] MamM-CTD, precipitated by zinc addition (blue) and the apo-protein precipitated using 2.2 M ammonium sulfate (red). Vertical dashed lines indicate imidazole resonances in the protein. (B) ^13^C NMR spectra of the same samples showing a shift in imidazole carbons (vertical dashed lines) and the presence of Trp247-C^γ^ carbon only in the zinc-precipitated protein (dotted line). (C-E) Slices of 2D ^13^C DARR spectrum of zinc-precipitated [U-^13^C, ^15^N] MamM-CTD with inter-subunit contacts shown in (C) and (E) and rigid zinc binding imidazole carbons indicated in (D) in accordance with spectrum in (B).

Further support for the conformational changes that MamM-CTD experiences upon cation binding was provided by ssNMR. The ^15^N and ^13^C CPMAS spectra (aromatic region) of [U-^13^C,^15^N] zinc-precipitate MamM-CTD and the apo-protein, precipitated from Tris buffer using 2.2 M ammonium sulfate solution (similar to the crystallization conditions which maintain apo-protein fold), are shown in Fig. 3A–B. The histidine imidazole region in the apo-protein shows several weak resonances (165–195 ppm) ascribed to histidine residues adopting different conformations. In the zinc-bound state, imidazole resonances converge into a single line at 175 ppm representing the N^δ1^ (in protonated state) and a smaller line at 215 ppm representing the N^ε2^ [27] which are indicative of zinc complexation by histidine [28]. Moreover, exact location of zinc coordination by histidine can be inferred from the imidazole carbon resonances in the aromatic region of the ^13^C CPMAS spectra of the zinc-precipitate (red) and apo (blue) protein, shown in Fig. 3B. The average chemical shift difference between the two imidazole carbons Δ_εδ_ = δ{C^ε1^}-δ{C^δ2^} is a sensitive tool of zinc coordination and, as recently shown, can be used to determine to which nitrogen atom the metal is coordinated [29]. A Δ_εδ_ of 12.32 ppm is attributed to binding via N^ε2^ and a value of 19.8 ppm to binding via N^δ1^. The C^δ2^ is located in the broad 127 ppm resonance and C^ε1^ is shifted from 135.5 ppm in the apo form to 137.4 ppm in the zinc-bound form, giving rise to a Δ_εδ_ of 10.4 ppm, which indicates that zinc complexation is mediated by the N^ε2^ nitrogen of histidine residues in MamM-CTD. In the ^13^C spectrum, the upfield resonance at 112.3 ppm – associated with Trp247 C^γ^ carbon – is only observed in the zinc-precipitate and may be attributed to restricted motion of the indole ring due to the involvement of Trp in binding, in accordance with the fluorescence and computational data. Evidence for the formation of intermolecular contacts between the two monomers in the hinge region is obtained from two-dimensional (2D) ^13^C Dipolar Assisted Rotational Resonance (DARR) [30] measurements carried out on the zinc-bound MamM-CTD, shown in Figs. 3C,3E. Cross-peaks in the DARR spectrum are unambiguously assigned to contacts between Thr259-C^β^ and Glu257-C and between Glu289-C^γ^ and His264-C^δ1^ (Fig. 3C–E). The imidazole carbons which are typically missing in such 2D spectra due to motions were observed (Fig. 3D), giving further support for restricted mobility due to their participation in metal binding, as discussed above. Overall, these results indicate that MamM-CTD undergoes conformational changes upon divalent cation binding towards a tighter packed dimeric structure.

### Characterization of (putative) metal-binding sites

**Figure 4:**
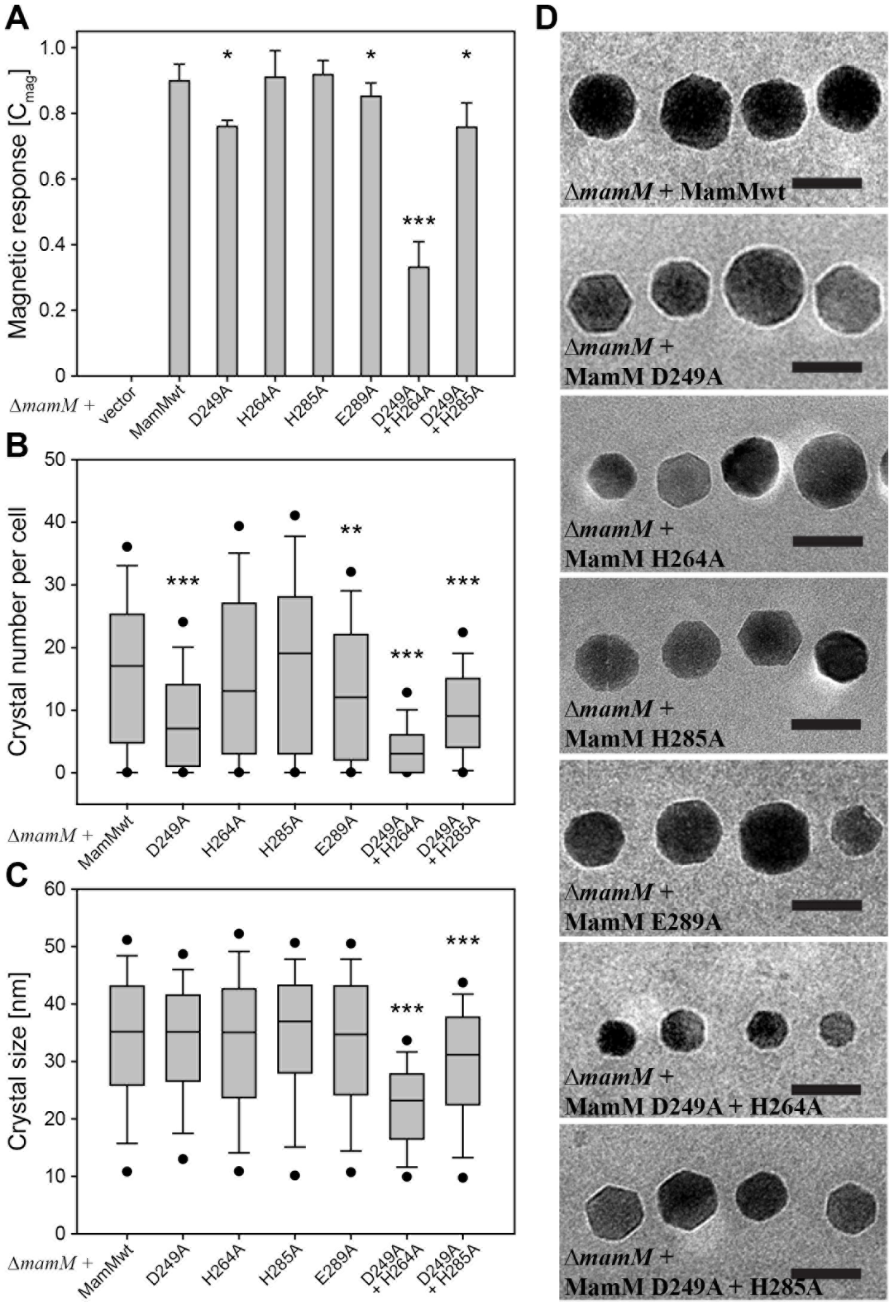
Effects of alanine substitutions within the putative MamM-CTD cation binding sites on magnetic response, crystal number per cell, crystal size and crystal shape. (A) Magnetic response of Δ*mamM* strains expressing wild-type *mamM* or cation binding site mutant derivatives. Values are given as means ± standard deviations from ≥9 independent cultures. Statistical significance of alterations from the strain expressing wild-type *mamM* was tested using the *t*-test (*, *P*<0.05; ***, *P*<0.001). (B) Box plot showing the distribution of crystal numbers per cell from Δ*mamM* strains expressing wild-type *mamM*, or cation binding site mutant derivatives. Statistical significance of alterations from the strain expressing wild-type *mamM* was tested using the Mann-Whitney test (**, *P*<0.01; ***, *P*<0.001). (C) Box plot showing the magnetite crystal size distribution of Δ*mamM* strains expressing wild-type *mamM*, or cation binding site mutant derivatives. Statistical significance of alterations from the strain expressing wild-type *mamM* was tested using the Mann-Whitney test (***, *P*<0.001). (D) Representative TEM images of magnetite crystals from Δ*mamM* strains expressing wild-type *mamM*, or cation binding site mutant derivatives. Scale bar, 50 nm.

To elucidate the roles of the three putative binding sites in our proposed regulatory divalent cation sensory domain, we tested the effect of alanine point mutations on magnetite biomineralization *in vivo*. In previous *in vivo* studies, deletion of the *mamM* gene entirely abolished magnetosome biomineralization, whereas transcomplementation of the MSR-1 ΔmamM strain with alleles carrying substitutions of various single amino acid residues resulted in smaller and fewer crystals, as indicated by the gradual decrease in cellular magnetic response (C_mag_) [25], [31]. In a similar manner, upon targeted mutagenesis of MamM, we monitored the resulting effects on magnetosome biomineralization as a sensitive probe of iron uptake by following changes of the Cmag as well as TEM analysis of the number, size and morphology of the formed electron-dense iron nanoparticles. Plasmid-derived transcomplementation of MSR-1 Δ*mamM* with the wild-type allele restored magnetosome formation to wild-type-like particle diameters (34±12 nm) but reduced particle numbers per cell by ∼50% (16±12 nm). As observed previously [25], the decreased number of particles per cell was at least partially a consequence of a substantial number of cells within the population that remained non-magnetic (10–20%). These previous studies [25] also revealed that transcomplementation of a truncated C-terminal tail MamM mutant also allows the formation of wild-type-like particle. Compared to transcomplementation with the wild-type allele, single point mutations of the histidine residues at the putative central binding site (H285A) or periphery binding sites (H264A) affected neither C_mag_ values nor the number, size and morphology of the crystals (Fig. 4; Fig. E in file S1, Table S3). In contrast, E289A from the peripheral binding sites resulted in a significantly lower magnetic response (Fig. 4A; Table S3) that correlated with a significantly lower number of magnetite particles per cell (Fig. 4B; Table S3; −17%; P<0.01, Mann-Whitney test). However, size and shape of the particles were unaffected in E289A mutants (Fig. 4C–D; Table S3). The central binding site D249A mutant also displayed a significantly decreased magnetic response (Fig. 4A; Table S3) that correlated with reduced numbers of magnetite particles per cell (Fig. 4B; Table S3; −47%; P<0.001, Mann-Whitney test). Although the average particle size in the cells expressing D249A mutant and in the cells expressing wild-type MamM was similar, the crystal size distribution of the D249A mutant showed a minor shift towards smaller crystals whereas no defects in crystal morphologies were observed (Fig. 4B–D; Table S3). Furthermore, only the D249A and E289A single mutants revealed an additional slight decrease of the magnetic response during iron induction experiments with iron-starved cells (Fig. F in file S1). ITC measurements of the CTD single point mutants and the C-terminal truncation mutant revealed that the binding affinities remained in the same order of magnitude as the wild-type, with some alternation in the number of binding sites. These ITC measurements are average values as a single site isotherm pattern was used to fit the experimental data and thus do not represent the individual Kd of each binding site (Table 1; Fig. D in file S1). The D249A, H285A and H264A mutants displayed a minor increase in the number of binding sites (Table 1) compared to the wild-type protein, which may be the result of an increased void volume that allowed the accommodation of additional cations after amino acid substitution to alanine. Whereas, only in the E289A mutant was the number of binding sites significantly reduced (2.0±0.2) (Table 1; Fig. D in file S1). Since Glu289 is located at the domain surface, it is more likely to have a role in peripheral binding site stabilization and metal ion coordination than the relatively buried His264. To gain a better understanding of the contribution of these binding sites to protein function two double alanine point mutations were also analyzed. *In vivo* the introduction of double point mutations in the central putative binding site (D249A and H285A) resulted in decreased C_mag_ values similar to D249A (Fig. 4A; Table S3). Although the average particle number per cell was almost identical between the D249A and the D249A + H285A strains (Fig. 4B), the latter strain produced particles with significantly reduced mean diameters (Fig. 4C; Table S3; −12%, *P*<0.001, Mann-Whitney test). In addition, this strain also showed a slower restoration of its magnetic response upon induction of biomineralization by adding iron back to iron-starved cells (Fig. F in file S1). ITC results for the central putative binding site double mutant (D249A and H285A) displayed a reduced number of binding sites (1.8±0.2) in reference to the wild-type data and therefore suggests that a single cation binds to each peripheral binding site. To test this hypothesis, we combined the D249A point mutation at the central binding site and the H264A mutation at peripheral sites. Although no cation binding was detected for the resulting double mutant, D249A + H264A, by ITC *in vitro* (Fig. D in file S1) it still produced magnetite particles *in vivo*. However, magnetite formation was severely affected in the D249A+H264A mutant as a significant non-additive decrease of the magnetic response, the average particle numbers and the average crystal sizes were observed (Fig. 4A–C; Table S3). In addition the D249A+H264A double mutant showed a drastically delayed formation of magnetite during iron induction experiments (Fig. F in file S1). These results suggest that proper sensory and regulatory activity of the CTD can be achieved through a single type of binding site, either the central or the two peripheral sites, to allow proper activation and transport of MamM TMD. To confirm that these observed phenotypic differences are due to true transport function alternations, we also examined the expression levels of all MamM mutants, their ability to stabilize MamB expression (Fig. F in file S1), performed MD simulations for dimer stability evaluation and determined their CTD structures (Table S1). As such, we found (i) a similar wild-type expression levels in all mutants (Fig. F in file S1). (ii) All mutants exhibited an apo-MamM CTD-like behavior along the MD simulations (see Fig. B in file S1 for representative MD simulations), and (iii) an absence of structural differences (Fig. A in file S1) was observed. These described that effects of mutations in the identified three putative binding sites may shed light on the significant role of the CTD in iron transport activation and regulation.

### Analysis of the highly stable dimerization interface

**Figure 5:**
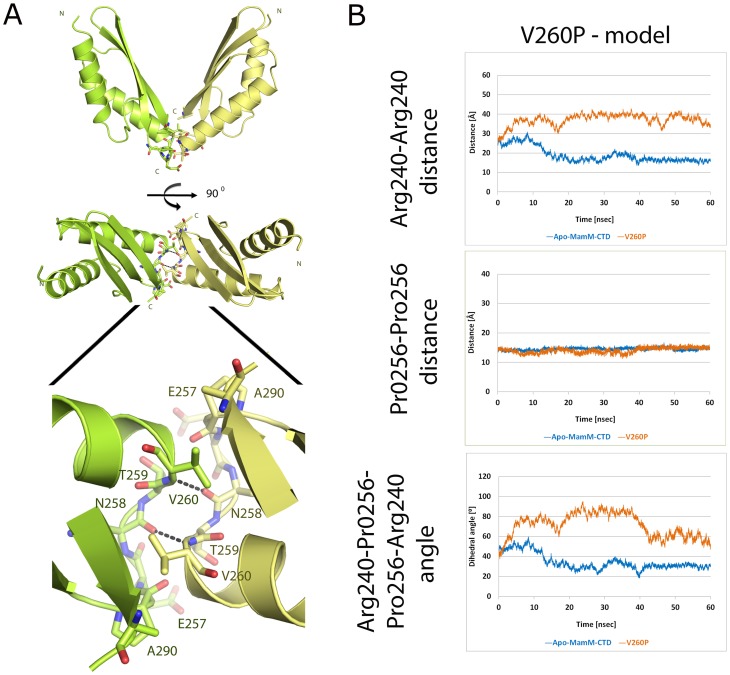
Dimerization interface of MamM-CTD. (A) The wild type dimeric fold is maintained by symmetrical backbone interactions between Asn258 and Thr259 and a single hydrophobic interaction between two symmetric Val260 giving rise to a dimerization interface surface of 193 Å^2^. (B) Molecular dynamic simulation analysis of V260P MamM-CTD mutant. The distances between the Cα of Arg240-Arg240, Pro256-Pro256 and the Cα Arg240-Pro256-Pro256-Arg240 dihedral angle of the apo-MamM-CTD structure with a modeled V260P mutation were monitored throughout the 60 nsec simulation (orange) and compared to wild type apo-MamM-CTD simulation (blue). To mimic the effect of TMD on the CTD a 60 Å N-terminals dimer distance restrain was inserted to the simulation. The V260P mutated apo-MamM-CTD model presents increased dihedral angle and Arg240-Arg240 distance in reference to the wild type MamM-CTD and maintain stable dimerization interface at the bottom of the V-shaped dimer.

MamM dimers present a single dimerization interface located at the bottom of the V-shaped fold. Val260 has a significant role in dimer stabilization, as the symmetrical interaction between two Val260 on two opposite monomers is the stand-alone hydrophobic interaction at the 193 Å^2^ dimerization interface (Fig. 5). To elucidate the role of this dimerization interface in CDF regulation we introduced an amino acid substitution in position 260 and examined its effect. A V260P mutation is predicted to alter the backbone hydrogen bonds between MamM monomers (Fig. 5). *In vitro* validation by size-exclusion chromatography revealed that recombinant MamM-CTD V260P remained a stable dimer in solution, suggesting that the overall protein fold was not altered by the mutation (Fig. 1A). According to the apo-MamM-CTD determined structure, a V260P structural model was constructed, minimized and subjected to MD simulation. This MD simulation was restrained by 60 Å maximum distance between the dimer's N–terminis to mimic the protein-related restrains provided by the dimeric TMD. Analysis of this restrained MD simulation also confirmed a stable dimer fold throughout the simulation with an increased dihedral angle and Arg240-Arg240 distance in reference to the wild-type MamM-CTD (Fig. E in file S1). Trans-complementation of MSR-1 *ΔmamM* cells with a *mamMV260P* allele demonstrated that magnetic behavior could not be restored and the cells remained non-magnetic (Table S3). In addition, ITC experiments revealed a significantly lower number of binding sites for the MamM-CTD V260P mutant and presented the lowest entropic contribution to the zinc binding (Table 1). Although the relatively small dimerization interface maintained by two polar interactions and single hydrophobic interaction it remains stable dimer even upon V260P mutation. However, a stable dimer is not sufficient to allow proper function of the protein as seen by the *in vivo* results.

By considering these results, the CTD movement can be described as a spring motion. The negatively charged V-like shaped "arms" are drawn apart due to charge repulsion whilst the hydrophobic interactions at the dimerization interface are pushing the "arms" closer towards a tolerable and favorable distance which maintains stable hydrophobic interactions (Fig. C in file S1). Hence, the CTD functionality depends on the subtle equilibrium between charge repulsion and hydrophobic interactions. Altogether these results suggest that the CTD's induced conformational shift upon cation binding provides the activation signal to the TMD. Furthermore, the presence of a small hydrophobic residue at the dimerization interface allows the proper dimer movement upon cation binding as it provides an additional stabilizing interaction for the ion transporting closed state.

## Discussion

Iron biomineralization by MTB is a highly complex biological process governed by multiple proteins that yield structurally perfect magnetite nano-crystals. One of the most important MTB proteins is MamM, a predicted magnetosomal iron transporter to the MM vesicles [25]. As CDF proteins share high structural and functional similarities, characterization of the magnetosomal MamM protein provides insights applicable to the CFD family in general. In this study we utilized multi-disciplinary approaches to analyze the function and to determine the role of the MamM cytosolic domain.

Four main highlights of our study are as follows. First, we demonstrated by several complementary techniques that the MamM-CTD adopts a typical CDF CTD fold and exists as a dimer even in the absence of divalent metal cations. These results contradict findings of a previous study which proposed that upon divalent cation release the CDF CTD monomers the dimerization interface located at the bottom of the V-shaped dimer is disrupted [16]. However, our *in vivo*, *in vitro* and computational results have demonstrated a stable dimerization interface that even endured the introduction of a Val to Pro mutation that is predicted to alter the backbone hydrogen bonds in the dimer. As the metal-free CTDs of the CDF transporter CzrB [18] and TM0876 [17] were also crystallized in a V-shaped dimeric state we suggest that CDF-CTDs permanently form highly stable dimers.

Second, using MD simulations we also provided evidence that the MamM-CTD dimer shows a high natural flexibility in solution. This CTD flexibility is based on two opposite forces; the first is the charge repulsion between the two monomers whilst the second is the attraction via hydrophobic interaction at the center of the internal dimerization interface, pulling the monomers closer. Due to these opposing forces and the protein-related restrains provided by N-terminal TMDs the CTD flexibility is converted into a spring-like motion. Interestingly, Trp fluorescence and SAXS measurements as well as MD simulations indicated that upon metal cation binding the flexibility of the MamM-CTD is restricted and a stable closed-state MamM-CTD is formed. Thus, we propose that the CTD movements of the apo-form are used to “search” for appropriate metal cations, which when bound to the CTD lead to a more static closed dimer.

Third, to analyze the role of metal cation binding for the overall function of the MamM transporter we performed detailed analyses of the MamM-CTD metal binding sites. However, as we were not able to generate metal-bound MamM-CTD crystals we first had to use an *in silico* approach to identify metal binding sites within MamM-CTD dimers. Using the metal-bound CzrB as a template we identified three metal binding sites on the dimer surface composed of the Asp249, His264, His285 and Glu289 residues of different monomers. Notably, MD simulation of this MamM closed-conformation model maintained the stable binding and coordination of four zinc cations by these identified putative binding residues. Although sharing binding coordinating His and Asp residues, MamM and CzrB do not share identical spatial locations of the metal binding sites. In good agreement with these predicted metal-coordinating residues we observed a pH dependent metal binding in ITC experiments, which also suggested the participation of histidine residues in metal cation binding. Fourth, we confirmed metal coordination by the His264 and His285 residues by ssNMR. As a *mamM* deletion strain is unable to form magnetite particles the *in vivo* effects of mutations within the identified metal binding sites were analyzed by transcomplementation assays and subsequent determination of the cellular magnetic response, size and number of magnetite crystals. These *in vivo* analyses revealed a reduced magnetite formation only in those mutants that were also impaired in ITC zinc binding experiments. All tested mutant proteins were able to interact with MamB as determined by wildtype-like MamB expression levels. Therefore, the reduced magnetite formation reflects decreased MamM activities caused by less (e.g. MamM D249A) and/or shorter (e.g. MamM D249A+H264A) TMD activation events. Thus, our results indicate that metal binding by the CTD mediates MamM transport activity and that each metal binding site on its own is able to sense metal cations and activate the TMD of MamM.

**Figure 6:**
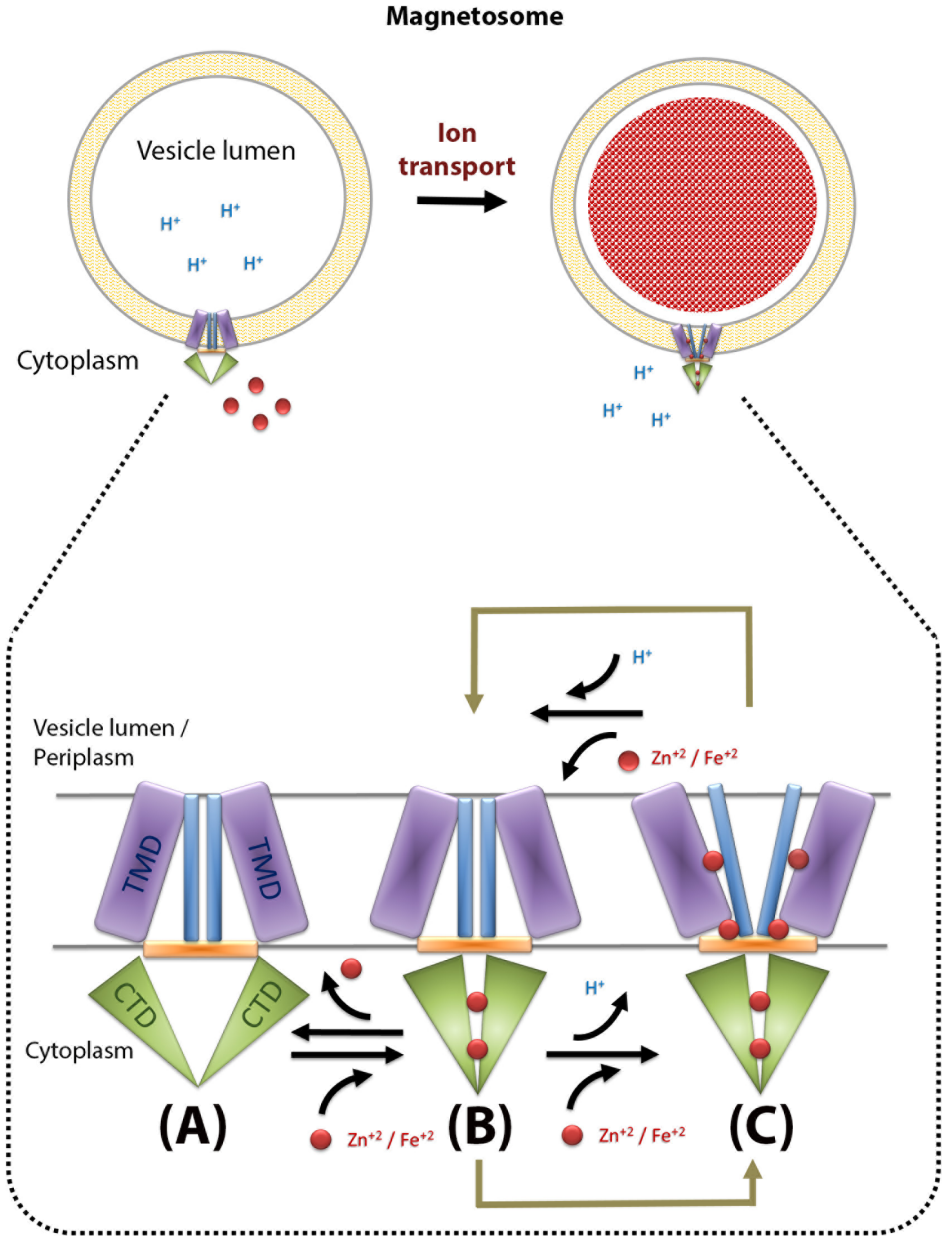
Proposed three-step transport mechanism of Cation Diffusion Facilitator (A–C). Upon sufficient cytoplasmic concentration the CTD binds divalent cations that trigger conformational changes toward a tighter and compact fold (A–B). This conformational change allows the activation of a two-step ion transport through the TMD. The TMD can then undergo alternate conformational changes during cation transport between cytoplasm-facing to vesicle lumen/ periplasm-facing conformations (B–C). Reduction of cytoplasmic divalent cation concentrations leads to ion dissociation from the CTD which induces the reconversion to open conformation and the completion of ion transport (C–A).

To summarize our results we present an alternative model for the CDF CTD mode of action (Fig. 6), in which we propose that upon metal cation binding, the CTD induces conformational changes towards a tighter and more compact fold which allows the activation of iron transport through the TMD. We presume that cation binding to the CTD is first mediated by a central site followed by secondary binding to the two peripheral binding sites to achieve further stabilization of the closed dimer. Recent electron microscopy reconstruction studies of a 13 Å resolution partial zinc-bound FieF homolog revealed that the TMD undergoes conformational changes from a cytoplasm-facing conformation upon insufficient cation concentrations to a periplasm-facing conformation upon cation transport [32]. Although the structure of this FieF homolog presents a novel TMD conformation which is presumed not to allow cation transport, the introduction of low zinc concentration during two-dimensional crystallization was sufficient to permit a closed and active conformation of the CTD. Hence, the FieF homologous model does not address the initial activation mechanism of the CTD by divalent cations but rather presents a two-step mechanism for the cation transport through the TMD (Fig. 6). In our model we suggest that the CTD undergoes an additional initiation step which later allows the two-step transport mechanism of the TMD. However, we cannot rule out a possible alternative CTD activation mechanism for FieF and homologous proteins.

Overall, our structural and biochemical data shed new light onto the mode of action of CDF proteins, especially on the regulatory sensing activity of their CTD. Furthermore, we promote new prospects on the initiation of CDF cation transport by in-depth analysis of the apo-MamM protein.

## Experimental Procedures

### Protein expression, purification and site-directed mutagenesis

Performed as previously described [33], [34].

### Crystallization and structure determinations

Crystallization and crystallographic statistics are in Tables S1 and S2. For full description see extended experimental procedures.

### Bacterial strains, oligonucleotides and plasmids for *in vivo* characterization

Bacterial strains, oligonucleotides, and plasmids used in this study are listed in Table S4. All strains were cultivated as described previously [35].

### Structure determination

Purified MamM-CTD and the mutants crystallized by vapor diffusion at different conditions at 20°C (Table S2). Crystals were harvested with addition of cryo-protecting solution and flash-cooled in liquid nitrogen. Data collections were performed at beamlines ID14-4 and ID23-2 at the European Synchrotron Radiation Facility (ESRF), Grenoble, France or at a home source marμX X-ray system (MarResearch, Germany) equipped with an image plate detector system (MAR 345 mm). Data were reduced and scaled using the HKL2000 [36] suite. MamM-CTD phases were obtained using Phaser molecular replacement and PDBcode: 3BYP as a template [18]. The final model was built by Coot [37] and refined in REFMAC [38]. For Rfree calculation, 5% of the data were excluded. Phases for all mutants structures were obtained by Phaser molecular replacement using the PDBcode: 3W6X. Structural figures were prepared with PyMOL [39].

### Least-squares overlaps

R.M.S. calculations were performed with SwissPDB viewer [40] using the domain alternate fit to align structures on the basis of the conserved domain and to define the conformational changes of the structural homologues.

### Electrostatic potential calculations

Electrostatic calculations were done in PyMOL [39] using the Adaptive Poisson-Boltzmann Solver (APBS) plug-in [41].

### Small angle X-ray scattering (SAXS) measurements

SAXS measurements of 0.42 mM MamM-CTD protein with or without zinc sulfate (17 μM) were performed using the SAXSLAB GANESHA 300-XL system with Cu Kα radiation generated by a sealed microfocused tube (Genix 3D Cu-source with integrated Monochromator) powered at 50 kV and 0.6 mA and three pinholes collimation. The scattering patterns were recorded by a Pilatus 300K detector. The scattering intensity I(q) was recorded in the interval 0.012<q<0.7 A-1, where q is defined as 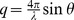, 2θ is the scattering angle and λ is the radiation wavelength (1.542 Å). The solution under study was sealed in a thin-walled capillary (glass) of about 1.5 mm diameter and 0.01 mm wall thickness. Measurements were performed under vacuum at a temperature of 4°C. The 2D SAXS images were azimuthally averaged to produce one-dimensional profiles of intensity, I vs. q, using the two-dimensional data reduction program SAXSGUI. The scattering spectra of the capillary and solvent were also collected and subtracted from the corresponding solution data. No attempt was made to convert the data to an absolute scale.

### SAXS data analysis and envelope model

The radius of gyration (Rg) was evaluated using the Guinier approximation [42]. The GNOM program was used to obtain Pair-distance distribution functions, corresponding maximum dimension of protein complexes (Dmax) and to determine the value for Rg from the entire scattering profile [43]. Ab-initio envelopes were generated by the program DAMMIN using atomic radii set to the dummy atom packing radius determined by DAMMIN without imposing symmetry operation [43]. The generated envelope models (DBMs) were fitted on the core X-ray determined wild type structure using the Coot software [37] and visualized by PyMOL [39].

### Trp fluorescence

Changes in Tryptophan intrinsic emission were measured for 0.84 μM protein solutions titrated with 24 mM zinc chloride or 219 μM ammonium ferric sulfate. Fluorescence emission spectra were acquired at 27°C on an Edinburgh FL920 spectrofluorimeter, using excitation at 280 nm. Total sample volumes were 1 mL and the solutions were placed in a quartz cell having a 1 cm optical path length. Light scattering from the buffer was confirmed to account for less than 1% of the emission intensity.

### Classical Molecular Dynamics (MD) simulations

MD simulations had been used to examine the stability of the apo and closed structures. Furthermore, the MD simulations may give insight into the dynamics of these structures and allow following the fluctuations in proteins. MD simulations of solvated MamM protein models (apo and closed structures) were performed in NPT ensemble at 1 atm and 310 K or 318 K using the NAMD [44] program with the CHARMM27 force-field [45], [46]. The protein models were explicitly solvated with a TIP3P water box with a minimum distance of 15 Å from any edge of the box to any protein atom. Long-range electrostatic interactions were calculated using the particle mesh Ewald method with a cut-off of 12.0 Å for all simulations. Counter ions (Na^+^ or Cl^−^) were added at random locations to neutralize the charge of the protein models. MD simulations' conditions (310 K or 318 K and 60 ns of timescales) were applied to test the stabilities of all the variant models.

#### Experiment-Based MamM Protein Models' Construction

We used the crystallography structures of the MamM-CTD for the molecular dynamics (MD) simulations for the open state and for the closed state. The closed state does not include zinc ions, therefore for the zinc-binding protein models we added the Zn^2+^ in the binding sites. We further used the crystallographic structure of the V260R mutant and we made mutations to the wild-type crystallographic structure for the open state. The constructed models were minimized before the MD simulations, as previously we have performed for Zn^2+^-Aβ oligomers [47].

#### Molecular dynamics (MD) simulations protocol

MD simulations of the solvated variant models of the protein with and without Zn^2+^ were performed in NPT (N, number of particles; P, pressure; and T, temperature) ensembles using the NAMD program [44] with the CHARMM27 force-field [45], [46] for 60 ns. The models were explicitly solvated with TIP3P water molecules [48], [49]. The Langevin piston method [44], [50], [51] with a decay period of 100 fs and a damping time of 50 fs was used to maintain a constant pressure of 1 atm. The temperature (310 K or 318 K) was controlled by Langevin thermostat with a damping coefficient of 10 ps^−1^ [44]. The short-range Van der Waals (VDW) interactions were calculated using the switching function, with a twin range cutoff of 10.0 and 12.0 Å. Long-range electrostatic interactions were calculated using the particle mesh Ewald method with a cut-off of 12.0 Å for all simulations [52], [53]. The equations of motion were integrated using the leapfrog integrator with a step of 2 fs. All initial variant models were energy minimized and then solvated in a TIP3P water box with a minimum distance of 15 Å from any edge of the box to any protein atom. Any water molecule within 2.5 Å of the protein was removed. Counter ions (Na^+^ or Cl^−^) were added at random locations to neutralize the protein's charge. The solvated systems were energy minimized for 2000 conjugated gradient steps. The counter ions and water molecules were allowed to move. The minimized solvated systems were heated at 200 K, where all atoms were allowed to move. The systems were then heated from 200 K to 250 K for 300 ps and equilibrated at 310 K or 318 K for 300 ps. All simulations ran for 60 ns and structures were saved every 10 ps for analysis. These conditions (310 K or 318 K and 60 ns of timescales) were applied to test the stabilities of all the variant models.

### Trans-complementation of ΔmamM

For trans-complementation assays pRU1 and *mamM* containing derivatives were transferred to Δ*mamM* by conjugation. After plasmid transfer the average magnetic response (C_mag_) of three independent trans-conjugants was assayed as described [31]. Imaging of trans-complemented cells by transmission electron microscopy (TEM) was performed as previously described [54]. Expression of *mamM* and site-directed variants was confirmed by separation of 10 μg of whole cell protein by SDS-polyacrylamide (12%) gel electrophoresis (PAGE) and subsequent Western blot analysis as previously described [25].

### Solid state NMR

Magic angle spinning NMR measurements were carried out on a 500 MHz BrukerAvanceIII spectrometer on a 4 mm CPMAS probe at a sample spinning rate of 10 kHz. Composite pulse decoupling using the SPINAL64 sequence was employed through acquisition in all measurements. ^15^N cross polarization experiments were carried out using a ^1^H 90° pulse of 3.2 μs, followed by a 5 ms^1^H to ^15^N polarization transfer time with a r.f. pulse on ^1^H channel ramped between 76 and 38 kHz and a r.f. pulse of 47 kHz on ^15^N with recycle delay of 2 s. ^13^C cross-polarization experiments were carried out using a ^1^H 90° pulse of 3.2 μs followed by a 2 ms proton-to-carbon polarization transfer time with a r.f. pulse on ^1^H channel ramped between 70 and 35 kHz and a r.f. pulse of 62 kHz on ^13^C with recycle delay of 2 s. 2D ^13^C DARR measurements employed similar excitation of carbon signal as in the CPMAS measurement and ^13^C 90° pulses of 5.7 μs using a ^1^H-^1^H mixing of 50 ms and similar recycle delay.

### Isothermal titration calorimetry

Isothermal titration calorimetry measurements were performed on an iTC200 calorimeter (Microcal, GE Healthcare) at 25°C. Both protein and zinc chloride were diluted to the same final buffer of 10 mM Tris·HCl, pH 8.0, 150 mM NaCl, or 10 mM MES, pH 5.7, 150 mM NaCl. Aliquots (1.8 μl) of the zinc chloride solution (5 mM) were titrated every 150 sec. The data were fit using ORIGIN 7.0 software (Origin Lab) to the single-site binding isotherm. The integrated peak of the first injection was excluded from the fit due to the large errors in the first step.

### Coordinates

Structures have been submitted to the Protein Data Bank (3W5X, 3W5Y, 3W5Z, 3W60, 3W61, 3W62, 3W63, 3W64, 3W65, 3W66, 3W8P).

## Supporting Information

Table S1 **Data collection and refinement statistics.**
(PDF)

Table S2 **Crystallization of MamM-CTD and mutants.**
(PDF)

Table S3 **Crystal number and size in trans-complemented Δ** ***mamM*** **cells.**
(PDF)

Table S4 **Bacterial strains, oligonucleotides and plasmids for in vivo characterization.**
(PDF)

File S1 **Supporting information figures.**
(PDF)
