# Supplementary material for: Cation Diffusion Facilitators Transport Initiation and Regulation Is Mediated by Cation Induced Conformational Changes of the Cytoplasmic Domain
Source: PLoS One. 2014 Mar 21;9(3):e92141. doi: 10.1371/journal.pone.0092141 (PMC3962391; doi:10.1371/journal.pone.0092141)
Supplement: Table S1 — Data collection and refinement statistics. (PDF) [file pone.0092141.s001.pdf]

Table S1 - Data collection and refinement statistics.

PDB code	3W5Y	3W5X	3W5Z	3W60	3W61	3W62
Protein	MamM CTD	MamM CTD	MamM CTD D249A	MamM CTD H264A	MamM CTD H285A	MamM CTD E289A
**Data collection**	ID14-4 - ESRF	ID14-4 - ESRF	ID23-2 - ESRF	ID23-2 - ESRF	ID23-2 - ESRF	ID23-2 - ESRF
Space group	F23	$C222_1$	$C222_1$	$C222_1$	$C222_1$	$C222_1$
Cell dimensions						
a, *b*, *c* (Å)	133.38,133.38,133.38	36.88, 97.56, 53.54	36.29, 94.79, 53.39	37.36, 95.12, 53.81	36.72, 94.91, 53.68	36.50,94.90,53.44
α, β, γ (°)	90, 90, 90	90, 90, 90	90, 90, 90	90, 90, 90	90, 90, 90	90, 90, 90
Resolution (Å)	1.952	1.602	1.65	1.82	1.59	1.64
*R*sym or *R*merge	6.2 (43.4)	5.8 (48.0)	3.8 (53.1)	5.0 (48.0)	4.0 (27.0)	4.3 (26.4)
I / σ*I*	43.62 (5.93)	48.68 (3.88)	59.58 (3.13)	31.70 (2.73)	40.28 (5.15)	35.72 (5.53)
Completeness (%)	96.1 (100)	98.2 (83.4)	99.8 (100)	98.6 (99.8)	99.6 (99.2)	99.5 (100)
Redundancy	5.6	6.1	8.8	5.8	6	5.9
Wavelength (Å)	0.939	0.939	0.873	0.873	0.873	0.873
**Refinement**						
Resolution (Å)	1.952	1.602	1.65	1.82	1.59	1.64
No. reflections	13839	12559	11245	8874	13139	11843
*R*work / *R*free	20.66/26.87	17.03/21.50	19.14/22.94	25.15/32.00	17.94/21.22	17.98/20.41
No. atoms						
Protein	1262	653	649	638	649	648
Ligand/ion	15	12	11	10	10	10
Water	102	82	55	52	78	77
B-factors						
Protein	37.13	24.07	28.63	33.07	23.57	24.86
Ligand/ion	61.60	22.90	30.17	33.70	19.42	21.58
Water	44.71	38.23	39.05	39.46	36.03	37.72
R.m.s. deviations						
Bond lengths (Å)	0.0232	0.0228	0.0254	0.0231	0.0254	0.0247
Bond angles (°)	1.8633	2.0303	1.8853	1.8124	1.7564	1.8552

PDB code	3W63	3W64	3W66	3W65	3W8P
Protein	MamM CTD 215-293	MamM CTD 215-293	MamM CTD D249A&H285A	MamM CTD D249A&H264A	MamM CTD D249A&H285A
**Data collection**	ID23-2 - ESRF	Home source	Home source	Home source	ID14-4 - ESRF
Space group	$C222_1$	$P2_12_12_1$	$C222_1$	$C222_1$	$C222_1$
Cell dimensions					
a, *b*, *c* (Å)	36.53, 94.86, 53.38	65.01,74.81,88.46	34.68, 93.87, 50.95	36.91, 94.48, 53.58	75.56, 87.53, 64.69
α, β, γ (°)	90, 90, 90	90, 90, 90	90, 90, 90	90, 90, 90	90, 90, 90
Resolution (Å)	1.9	2.85	2.05	2.37	1.8
*R*sym or *R*merge	9.8 (41.4)	12.6 (49.4)	5.9 (32.4)	7.1 (40.8)	8.7 (50.1)
I / σ*I*	21.7 (4.46)	17.42 (4.00)	33.74 (3.97)	31.13 (3.98)	41.53 (6.38)
Completeness (%)	99.8 (100)	99.7 (99.8)	97.9 (84.3)	99.9 (99.0)	99.4 (100)
Redundancy	6.4	6.7	7.7	8.1	10.4
Wavelength (Å)	0.873	1.5418	1.5418	1.5418	0.947
**Refinement**					
Resolution (Å)	1.9	2.85	2.05	2.37	1.8
No. reflections	7854	10553	5384	4059	19801
*R*work / *R*free	17.88/23.04	22.30/26.96	18.17/24.24	19.11/24.47	20.67/24.60
No. atoms					
Protein	638	2510	665	643	1441
Ligand/ion	10	48		10	20
Water	90	38	29	31	166
B-factors					
Protein	23.93	23.62	39.71	29.64	34.68
Ligand/ion	26.31	27.74		39.36	41.72
Water	33.55	21.12	39.13	39.88	40.74
R.m.s. deviations					
Bond lengths (Å)	0.0238	0.0174	0.017	0.0137	0.0184
Bond angles (°)	1.8568	1.6953	1.9026	1.7131	1.9759

Values in parentheses are for the highest resolution shell. One crystal was used per data set. Data was collected at 100 K for all crystals.

Data collections were performed at beamlines ID14-4 and ID23-2 at the European Synchrotron Radiation Facility (ESRF), Grenoble, France or at a home source marμX X-ray system (MarResearch, Germany) equipped with an image plate detector system (MAR 345 mm).
