# Supplementary material for: Cation Diffusion Facilitators Transport Initiation and Regulation Is Mediated by Cation Induced Conformational Changes of the Cytoplasmic Domain
Source: PLoS One. 2014 Mar 21;9(3):e92141. doi: 10.1371/journal.pone.0092141 (PMC3962391; doi:10.1371/journal.pone.0092141)
Supplement: Table S2 — Crystallization of MamM-CTD and mutants. (PDF) [file pone.0092141.s002.pdf]

Table S2 - Crystallization of MamM-CTD and mutants.

PDB code	3W5Y	3W5X	3W5Z	3W60	3W61	3W62	3W63
Protein	MamM CTD	MamM CTD	MamM CTD D249A	MamM CTD H264A	MamM CTD H285A	MamM CTD E289A	MamM CTD 215-293
**Data collection**	ID14-4 - ESRF	ID14-4 - ESRF	ID23-2 - ESRF	ID23-2 - ESRF	ID23-2 - ESRF	ID23-2 - ESRF	ID23-2 - ESRF
Space group	F23	$C222_1$	$C222_1$	$C222_1$	$C222_1$	$C222_1$	$C222_1$
Condition	7.5% 2-propanol 1.75M AmSO4	25% PEG 4,000 0.2M Ammonium sulfate 0.1M Sodium acetate	0.1M MES pH= 5.2 20% PEG 4000, 0.15M AmSO4	0.1M BIS-TRIS PH=5.5, 15% PEG 3350, 0.2M AmSO4	0.1M BIS-TRIS PH=5.5, 25% PEG 3350, 0.2M LiSO4	33% PEG 4000, 0.25M AmSO4	0.1M BIS-TRIS PH=5.5, 25% PEG 3350, 0.2M LiSO4
Cryo protctant	4M AmSO4	50% PEG 4,000	50% PEG 3350	50% PEG 3350	50% PEG 3350	50% PEG 4,000	50% PEG 3350
Protein concentration (mg/ml)	16	16	35	35	35	32	35
Crystallization type	Vapor diffusion sitting drop	Vapor diffusion sitting drop	Vapor diffusion sitting drop	Vapor diffusion sitting drop	Vapor diffusion sitting drop	Vapor diffusion sitting drop	Vapor diffusion sitting drop
Ramachandran statistics$^{\Phi}$	A: 150 (98.68%)	A: 71 (98.61%)	A: 73 (98.65%)	A: 71 (95.95%)	A: 72 (97.30%)	A: 70 (98.59%)	A: 72 (98.63%)
	P: 2 (1.32%)	P: 1 (1.39%)	P: 1 (1.35%)	P: 3 (4.05%)	P: 2 (2.70%)	P: 1 (1.41%)	P: 1 (1.37%)
	D: 0	D: 0	D: 0	D: 0	D: 0	D: 0	D: 0
Missing residues	A211-213 A294-318, B212, B293-318	211, 292-318	211-212, 293-318	211-212, 293-318	211-212, 293-318	211-212, 292-318	211-212, 292-318

PDB code	3W64	3W66	3W65	3W8P
Protein	MamM CTD 215-293	MamM CTD D249A& H285A	MamM CTD D249A& H264A	MamM CTD D249A& H285A
**Data collection**	Home source	Home source	Home source	ID14-4 - ESRF
Space group	$P2_12_12_1$	$C222_1$	$C222_1$	$C222_1$
Condition	0.1M BIS-TRIS PH=6.9, 25% PEG 3350, 0.2M LiSO4	0.1M BIS-TRIS PH=5.5, 25% PEG 3350, 0.2M AmSO4	0.1M BIS-TRIS PH=5.5, 25% PEG 3350, 0.2M AmSO4	0.1M BIS-TRIS PH=6.2, 26% PEG 3350, 0.2M AmSO4
Cryo protctant	50% PEG 3350	50% PEG 3350	50% PEG 3350	50% PEG 3350
Protein concentration (mg/ml)	35	35	28	35
Crystallization type	Vapor diffusion sitting drop	Vapor diffusion sitting drop	Vapor diffusion sitting drop	Vapor diffusion sitting drop
Ramachandran statistics$^{\Phi}$	A: 301 (96.78%)	A: 75 (97.40%)	A: 76 (96.2%)	A: 167 (100%)
	P: 10 (3.22%)	P: 2 (2.60%)	P: 2 (2.53%)	P: 0
	D: 0	D: 0	D: 1 (1.27%)	D: 0
Missing residues	A292-318, B211-212, B294-318, C211-213, C292-318, D292-318	294-318	294-318	A302-318, B211, B302-318

(Φ) A- Fully-allowed region, P- Partially-allowed region and D- Disallowed region

PEG- Polyethylene glycol.
