# Supplementary material for: Cation Diffusion Facilitators Transport Initiation and Regulation Is Mediated by Cation Induced Conformational Changes of the Cytoplasmic Domain
Source: PLoS One. 2014 Mar 21;9(3):e92141. doi: 10.1371/journal.pone.0092141 (PMC3962391; doi:10.1371/journal.pone.0092141)
Supplement: Table S3 — Crystal number and size in trans-complemented Δ mamM cells. (PDF) [file pone.0092141.s003.pdf]

Table S3 - Crystal number and size in trans-complemented Δ*mamM* cells

Parameter	Δ*mamM* (pRU1-mamMx)						
	WT	D249A	H264A	H285A	E289A	D249A-H264A	D249A-H285A
**Crystal number per cell**							
sample size	362	519	373	362	493	224	172
mean value	16.3	8.6	15.9	17.7	13.5	4.0	9.6
standard deviation	12.4	8.4	13.6	14.0	11.4	4.1	7.0
**Crystal size [nm]**							
sample size	1452	1723	946	732	1304	541	867
mean value	33.8	33.5	33.3	34.6	33.2	22.3	29.6
standard deviation	12.1	10.9	12.7	11.9	12.4	7.4	10.4
