# Supplementary material for: Cation Diffusion Facilitators Transport Initiation and Regulation Is Mediated by Cation Induced Conformational Changes of the Cytoplasmic Domain
Source: PLoS One. 2014 Mar 21;9(3):e92141. doi: 10.1371/journal.pone.0092141 (PMC3962391; doi:10.1371/journal.pone.0092141)
Supplement: Table S4 — Bacterial strains, oligonucleotides and plasmids for in vivo characterization. (PDF) [file pone.0092141.s004.pdf]

Table S4 – Bacterial strains, oligonucleotides and plasmids for in vivo characterization.

Strain/Oligo/Plasmid	Important features/Sequence	Source or reference
E. coli		
DH5α	F' Φ80d*lac* ΔM15 Δ(*lacZYA*-*argF*)U169 *deo*R *rec*A1 *end*A1	Invitogen
BW29427	*thrB1004 pro thi rpsL hsdS lac*ZΔM15 RP4-1360 Δ(*araBAD*)567 Δ*dapA1341::[erm pir* (wt)]	Obtained from Datsenko, K. and Wanner B. L.
M. gryphiswaldense		
MSR-1 R3/S1	Rif^r, Sm^r spontaneous mutant, wildtype	[1]
Δ*mamM*	R3/S1 but Δ*mamM*	[2]
**Oligonucleotide**		
MamMD249A_for	ACATCTGGGCCGCCATGATTATT	this study
MamMD249A_rev	CTTGGCCCACATAGCGAGCC	this study
MamMH264A_for	TCCCTGGCTGTCAGCGCAGAAGC	this study
MamMH264A_rev	TTCGATGCGGCGTATCTTTCCGC	this study
MamMH285A_for	CAGGCGGCTGAAATTTGCGAGGC	this study
MamMH285A_rev	TTCCACGGTGTTCTCGGGATCGACG	this study
MamMD289A_for	AGCGCAGCAGCGCGCGAAATC	this study
MamMD289A_rev	GACATGCAGGGATTCGATGCGGC	this study
MamMV260P_for	AACACCCCGGAACAGGCGCAT	this study
MamMV260P_rev	CTCGGGATCGACGCCAATAATCATG	this study
**Plasmid**		
pRU1	pBBR1MCS-2 with P_{mamAB}	(Uebe *et al.*, 2011)
pRU1-mamMwt	pRU-1 + *mamM*	(Uebe *et al.*, 2011)
pRU1-mamMD249A	pRU-1 + *mamM D249A*	this study
pRU1-mamMH264A	pRU-1 + *mamM H264A*	this study
pRU1-mamMH285A	pRU-1 + *mamM H285A*	this study
pRU1-mamMD289A	pRU-1 + *mamM D289A*	this study
pRU1-mamMD249A/H264A	pRU-1 + *mamM D249A + H264A*	this study
pRU1-mamMD249A/H285A	pRU-1 + *mamM D249A + H285A*	this study
pRU1-mamMV260P	pRU-1 + *mamM V260P*	this study
