# Supplementary material for: Cation Diffusion Facilitators Transport Initiation and Regulation Is Mediated by Cation Induced Conformational Changes of the Cytoplasmic Domain
Source: PLoS One. 2014 Mar 21;9(3):e92141. doi: 10.1371/journal.pone.0092141 (PMC3962391; doi:10.1371/journal.pone.0092141)
Supplement: File S1 — Supporting information figures. (PDF) [file pone.0092141.s005.pdf]

# Supporting Information for:

# Cation Diffusion Facilitators Transport Initiation and Regulation is Mediated by Cation Induced Conformational Changes of the Cytoplasmic Domain

**Natalie Zeytuni[*, †, ‡], René Uebe[¶¶, ‡], Michal Maes[**], Geula Davidov[*, †], Michal Baram[‡, ¶], Oliver Raschdorf [8], Merav Nadav-Tsubery[§§], Sofiya Kolusheva[¶], Ronit Bitton[§, ¶], Gil Goobes[§§], Assaf Friedler[**], Yifat Miller[§, ¶], Dirk Schüler[¶¶] and Raz Zarivach[*, †, #].**

[*]Department of Life Sciences and [†]National Institute for Biotechnology in the Negev, Ben Gurion University of the Negev, P.O.B. 653, Beer-Sheva 84105, Israel.

[‡]Department of Chemistry, Ben Gurion University of the Negev, P.O.B. 653, Beer-Sheva 84105, Israel.

[§]Department of Chemical Engineering Ben Gurion University of the Negev, P.O.B. 653, Beer-Sheva 84105, Israel.

[¶]Ilze Katz Institute for Nanoscale Science &Technology, Ben Gurion University of the Negev, P.O.B. 653, Beer-Sheva 84105, Israel.

[**]Institute of Chemistry, the Hebrew University of Jerusalem, Givat Ram, Jerusalem 91904 Israel.

[§§]Department of Chemistry, Bar-Ilan University, Ramat Gan 52900, Israel.

[¶¶]Ludwig Maximillian University Munich, Dept. Biology I, Großhaderner Str. 2, D-82152 Martinsried, Germany.

[‡]These authors contributed equally to this work.

[#]Correspondence should be addressed to Raz Zarivach, Department of Life Sciences, Ben Gurion University of the Negev, P.O.B. 653, Beer-Sheva 84105, Israel. Tel: +972-8-6461999, Fax: +972-8-6472970, Email: zarivach@bgu.ac.il

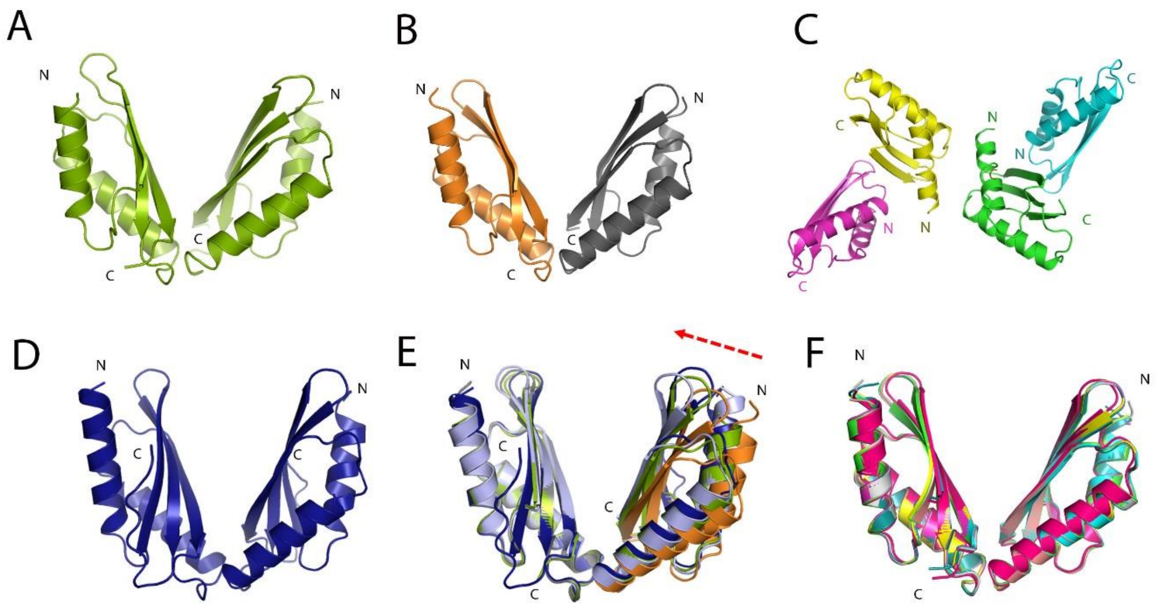


**<u>Fig. A</u>** – MamM-CTD and mutants crystal asymmetric unit composition and comparison. PDB codes: *(A)* 3W5Y – Wild type, *(B)* 3W5X – Wild type, monomer in orange, symmetry related monomer in gray, *(C)* 3W64 – C-terminal truncation mutant; residues 215-293, *(D)* 3W8P – D249A-H285A double mutant, *(E)* Overlay of the different dimeric forms from panels A-D presents a flexible movement at the N-terminal region, marked by a red arrow, which affects the maximal dimer diameter. *(F)* Overlay of MamM-CTD wild type and mutants crystallized in identical crystal lattice as presented in panel B confirms that mutations do not alter dimeric fold, PDB codes: 3W5X – Wild type, 3W5Z – D249A mutant, 3W60 – H264A mutant, 3W61 – H285A mutant, 3W62 – E289A mutant, 3W63 - C-terminal truncation mutant, 3W65 - D249A-H264A double mutant, 3W66 - D249A-H285A double mutant.

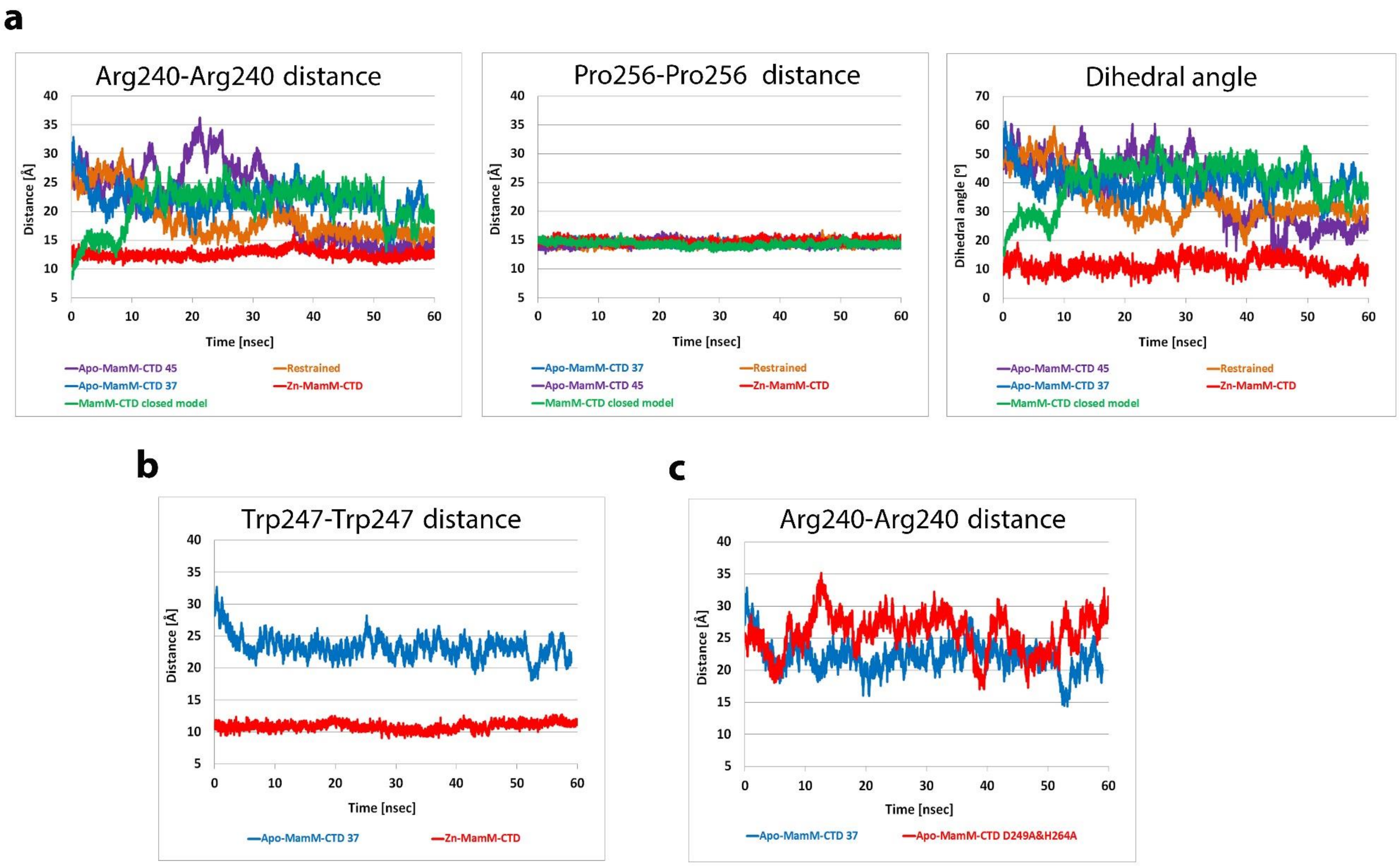

a
Arg240-Arg240 distance
Distance [Å]
Time [nsec]
Apo-MamM-CTD 45
Apo-MamM-CTD 37
MamM-CTD closed model
Restrained
Zn-MamM-CTD
Pro256-Pro256 distance
Distance [Å]
Time [nsec]
Apo-MamM-CTD 37
Apo-MamM-CTD 45
MamM-CTD closed model
Restrained
Zn-MamM-CTD
Dihedral angle
Dihedral angle [°]
Time [nsec]
Apo-MamM-CTD 45
Apo-MamM-CTD 37
MamM-CTD closed model
Restrained
Zn-MamM-CTD
b
Trp247-Trp247 distance
Distance [Å]
Time [nsec]
Apo-MamM-CTD 37
Zn-MamM-CTD
c
Arg240-Arg240 distance
Distance [Å]
Time [nsec]
Apo-MamM-CTD 37
Apo-MamM-CTD D249A&H264A

**<u>Fig. B –</u>** Molecular dynamic simulations analyses. *(A)* Five different simulations of wild type MamM-CTD: Apo-MamM-CTD in 37ºC, blue; Apo-MamM-CTD in 45ºC, purple; a maximal 60 Å N-terminal distance restrain of apo-MamM-CTD to mimic the transmembrane domain restrain in 37ºC, orange; four zinc ions bound MamM-CTD closed model in 37ºC, red; MamM-CTD closed model without zinc ions in 37ºC, green. *Left* - Distance fluctuations between the Cα of Arg240-Arg240 suggest that this region undergoes distinct conformational changes upon ligand binding. *Middle* - Distance fluctuations between the Cα of Pro256-Pro256 suggest that this dimerization region is highly stable and rigid. *Right* – Cα Arg240-Pro256-Pro256-Arg240 dihedral angle fluctuations demonstrate that the conformational changes upon ligand binding include ~35º twist. *(B)* Distance fluctuations between the Cα of Trp247-Trp247 suggest that this region undergoes distinct conformational changes upon ligand binding. *(C)* Representative simulation analysis for the MamM-CTD mutants. The distance fluctuations between the Cα of Arg240-Arg240 are similar for both wild type and D249A-H264A double mutant.

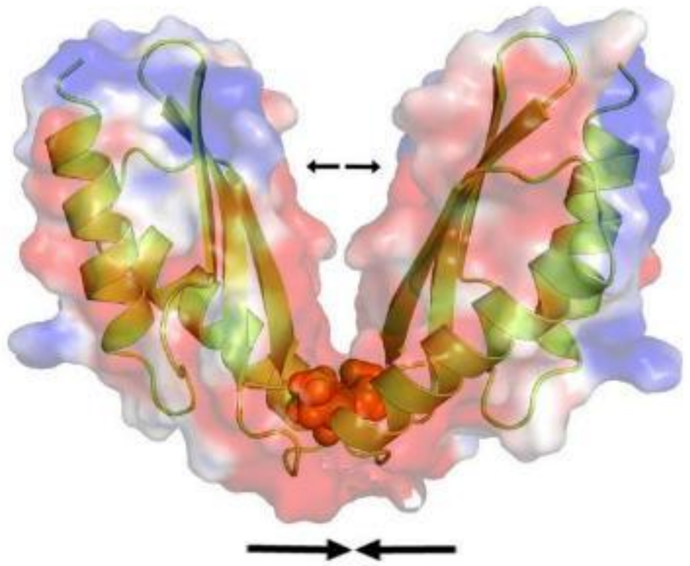

**<u>Fig. C</u>** – MamM-CTD functionality depends on the subtle equilibrium between charge repulsion and hydrophobic interactions and can be described as spring motion. Electrostatic surface representation of apo-MamM-CTD present a highly negatively charged surface at the center of the V-like shape dimer that is likely to be drawn apart due to charge repulsion. Hydrophobic interactions at the dimerization interface (symmetric Val260 residues) in sphere representation, are pushing the dimer closer toward a tolerable and favorable distance which maintains stable hydrophobic interactions.

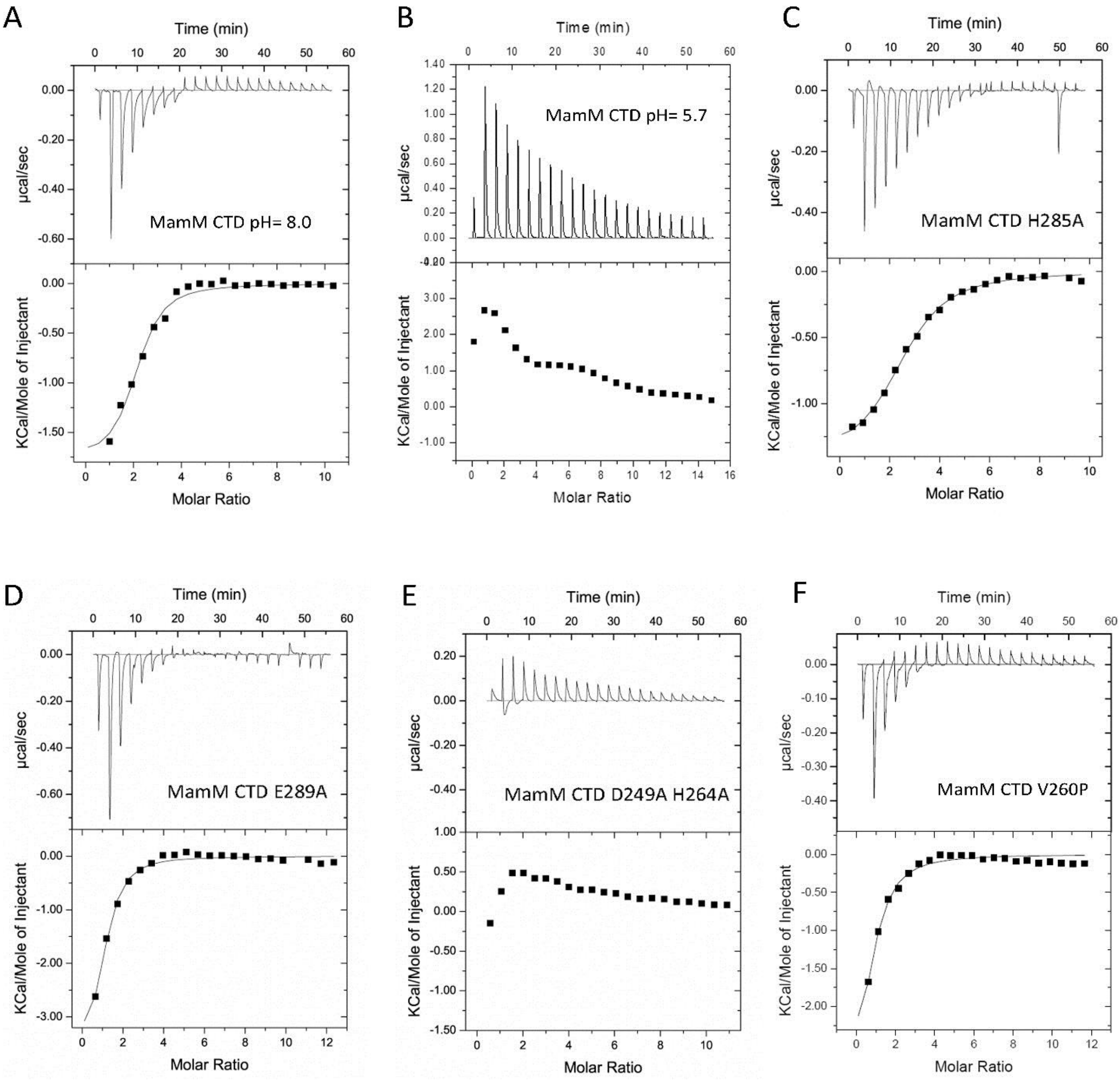


**Fig. D** - $Zn^{2+}$ pH dependent binding of the MamM-CTD**.** $ZnCl_2$ (5 mM) was titrated into: *(A)* MamM-CTD (50 µM), *(B)* MamM-CTD (68 µM) *(C)* MamM-CTD H285A (53 µM), *(D)* MamM-CTD E289A (42 µM) or *(E)* MamM-CTD D249A-H264A (47 µM) *(F)* MamM-CTD V260P (52 µM) in 1.8-µl aliquots every 150 s. *(A,C-F)* Measurements were performed at 25°C in 10 mM Tris·HCl pH 8.0, 150 mM NaCl. *(B)* Measurements were performed at 25°C in 10 mM MES pH 5.7, 150 mM NaCl. Top panels show the heat change during injection and bottom panels represent the data after peak integration. Data were fit using the Origin™ software to the single-site binding isotherm. See Table 1 for the binding parameters.

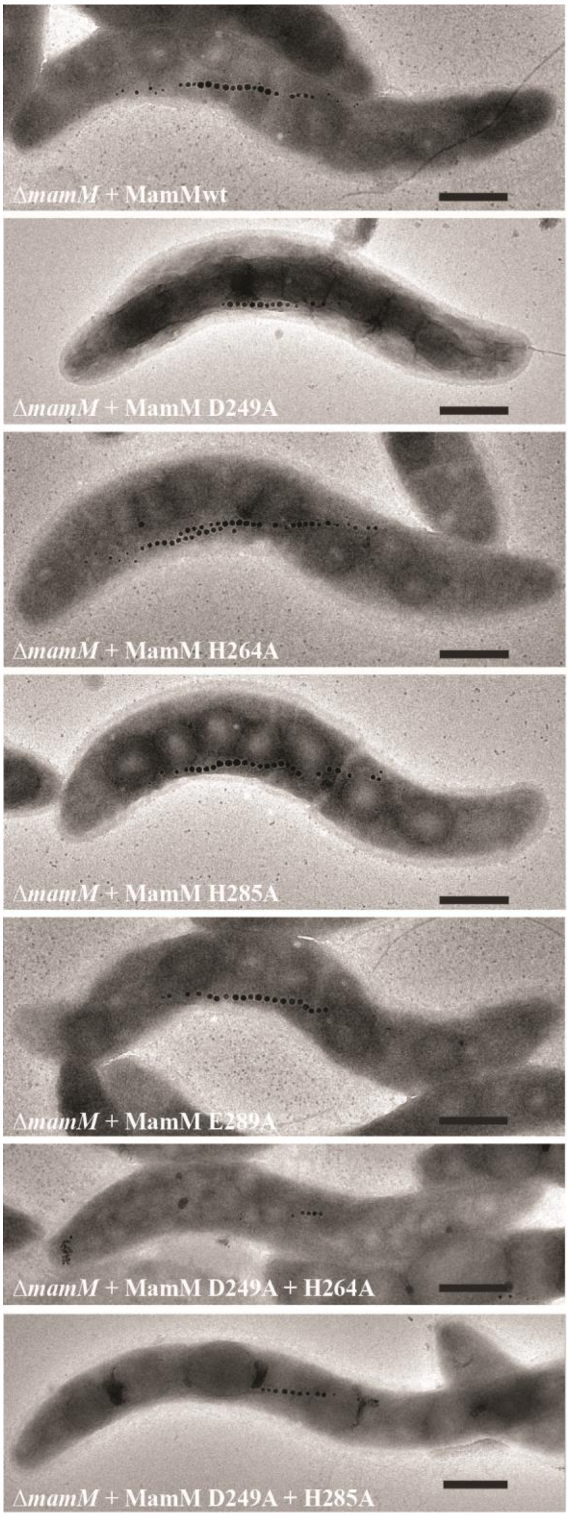


**<u>Fig. E</u>** - Representative TEM images of trans-complemented Δ*mamM* cells expressing wild type *mamM* or *mamM* with indicated mutations. Scale bars, 500 nm.

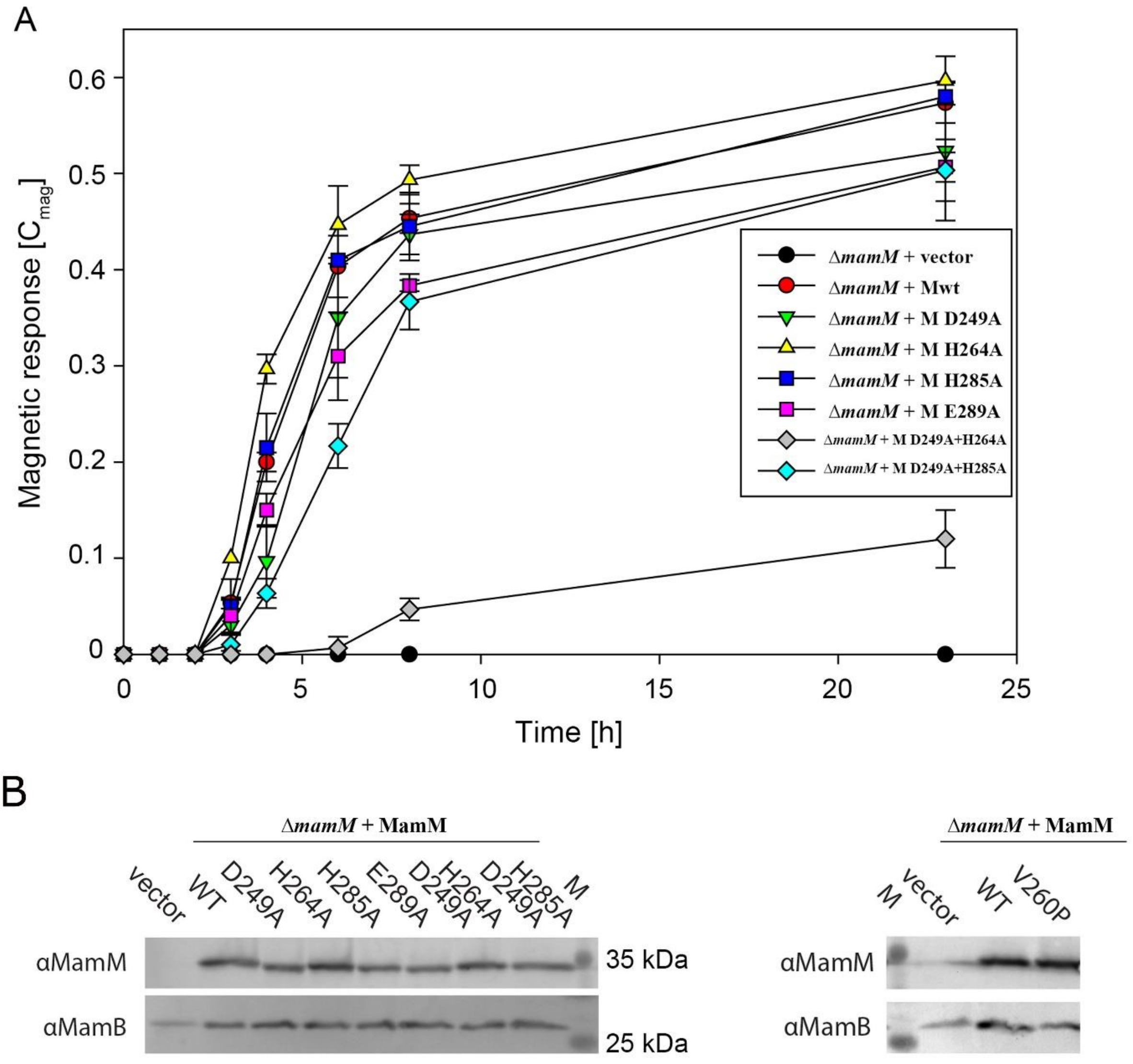


**Fig. F** - *(A)* Time-course of increase in magnetic response of trans-complemented Δ*mamM* strains. Cells were passaged in the absence of iron three times and then transferred into iron-containing media to induce magnetite crystal formation. The assay was performed in triplicate with three independent trans-conjugants and values are expressed as means, with standard deviations displayed as error bars. *(B)* Immunodetection of MamM and MamB wild-type and mutant proteins in cell lysates of trans-complemented Δ*mamM* strains. M, Molecular weight marker.
